# Protective Effect of Moderate Hypotonic Fluid on Organ Dysfunction *via* Alleviating Lethal Triad Following Seawater Immersion With Hemorrhagic Shock in Rats

**DOI:** 10.3389/fphys.2022.827838

**Published:** 2022-02-04

**Authors:** Yu Zhu, Haoyue Deng, Han She, Yuanqun Zhou, Yue Wu, Jie Zhang, Liangming Liu, Li Tao

**Affiliations:** State Key Laboratory of Trauma, Burns and Combined Injury, Shock and Transfusion of Research Institute of Surgery, Daping Hospital, Army Medical University, Chongqing, China

**Keywords:** seawater immersion, hemorrhagic shock, coagulation function, organ function, mitochondrial function

## Abstract

Previous studies found that seawater immersion combined with hemorrhagic shock (SIHS) induced serious organ function disorder, and lethal triad was a critical sign. There were no effective treatments of SIHS. Fluid resuscitation was the initial measurement for early aid following hemorrhagic shock, while the proper fluid for SIHS is not clear. Effects of different osmotic pressures [lactated Ringer’s (LR) solution, 0.3% saline, 0.6% saline, and 0.9% normal saline] on the lethal triad, mitochondrial function, vital organ functions, and survival were observed following SIHS in rats. The results showed that SIHS led to an obvious lethal triad, which presented the decrease of the body temperature, acidosis, and coagulation functions disorder in rats. Fluid resuscitation with different osmotic pressures recovered the body temperature and corrected acidosis with different levels; effects of 0.6% normal saline were the best; especially for the coagulation function, 0.6% normal saline alleviated the lethal triad significantly. Further studies showed that SIHS resulted in the damage of the mitochondrial function of vital organs, the increase of the vascular permeability, and, at the same time, the organ function including cardiac, liver, and kidney was disordered. Conventional fluid such as LR or 0.9% normal saline could not improve the mitochondrial function and vascular leakage and alleviate the damage of the organ function. While moderate hypotonic fluid, the 0.6% normal saline, could lighten organ function damage *via* protecting mitochondrial function. The 0.6% normal saline increased the left ventricular fractional shortening and the left ventricular ejection fraction, and decreased the levels of aspartate transaminase, alanine transferase, blood urea nitrogen, and creatinine in the blood. The effects of fluids with different osmotic pressures on the mean arterial pressure (MAP) had a similar trend as above parameters. The survival results showed that the 0.6% normal saline group improved the survival rate and prolonged the survival time, the 72 h survival rate was 7/16, as compared with the LR group (3/16). The results indicate that appropriate hypotonic fluid is suitable after SIHS, which alleviates the lethal triad, protects the mitochondrial function and organ functions, and prolongs the survival time.

## Introduction

Hemorrhagic shock is a comprehensive syndrome of the dysfunction of the systemic microcirculation, which led to serious impairment of vital organs due to the decrease of effective circulating blood volume and insufficient perfusion of tissue following traumatic injury (Mauriz et al., 2007; Torres Filho, 2017). Hemorrhagic shock was the main inducer in the death of patients with war trauma, accounting for 66–80% of all deaths (Li et al., 2012). With the maritime performance becoming frequent, sea immersion combination with injury presented more. At the same time, maritime military activities were increasing. Hemorrhagic shock in combination with seawater immersion was a crisis problem during military training and operations at sea. Seawater is a high-permeability, alkaline, and low-temperature liquid (Shen et al., 2017). Previous reports demonstrated that there was a series of pathophysiological characteristics following seawater immersion injury, such as microcirculation disturbance, inflammatory reaction, tissue and cell swelling, electrolyte imbalance, and body resistance decline (Wang et al., 2015; Yi et al., 2020). Our preliminary studies found that several pathophysiological parameter disorders were presented following the seawater immersion combined with hemorrhagic shock (SIHS), such as high incidence of the lethal triad and dysfunction of multiple organs, and electrolyte disturbance, which led to high mortality and brought large challenges to the emergency treatment.

Fluid resuscitation was the initial and key measurement for early treatment of traumatic hemorrhagic shock due to blood loss. Until present, resuscitation fluids that are commonly applied clinically include crystal fluid and colloid fluid. The crystal liquid mainly composed of lactated Ringer’s (LR) solution, compound sodium chloride, and the colloidal liquid mainly composed of hydroxyethyl starch (HES) and gelatin (Chipman et al., 2020). Previous studies found that seawater immersion induced the increase of osmotic pressure in blood circulation; as both LR and HES were isotonic liquids, it was not clear which osmotic pressure liquid was suitable for resuscitation following SIHS.

To elucidate this issue, with the rat model of seawater immersion combined with traumatic hemorrhagic shock, the effects of fluids with different osmotic pressures (0.9, 0.6, 0.3% salt water, and LR) on animal survival, the lethal triad, mitochondrial function, and major organ function were observed in this study. The main purpose was to look for a suitable fluid for the treatment of marine battle injuries.

## Materials and Methods

### Ethical Approval of the Study Protocol

All procedures were performed as per the guidelines for the Care and Use of Laboratory Animals published by the US National Institutes of Health. This study was approved by the Research Council and Animal Care and Use Committee of the Research Institute of Surgery, Daping Hospital, Army Military Medical University. None of the authors are members of this committee.

### Animal Preparation and Model Establishment

One hundred and ninety-two male Sprague-Dawley (SD) rats (220–240 g) received no food for 12 h but were allowed water *ad libitum* before the experiment. On the day of the experiment, rats were anesthetized with 30 mg/kg sodium pentobarbital. Two catheters were inserted into the right carotid artery and jugular vein for monitoring the mean artery pressure (MAP), blood volume, and fluid infusion. The temperature probe was placed near to carotid artery for monitoring the core body temperature. To prevent clotting, the carotid artery was filled with 500 U/kg of heparin sodium.

### Experimental Protocol and Phase

**FIGURE 1:**
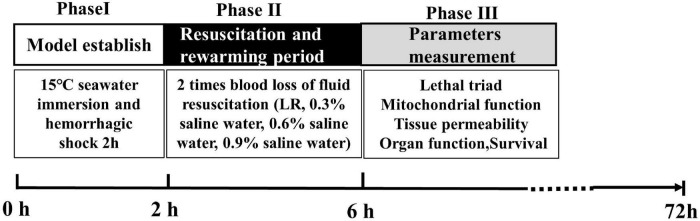
Timeline of the experimental phases.

Experiments were classified into four phases. Phase I was the SIHS period. During phase I, rats were immersed in artificial seawater at 15°C, and the seawater concentration was 2.535%. After immersion for 30 min, blood was drawn from the carotid artery until the blood loss reached 40% of the total blood loss volume. The total immersion time was 2 h, and the SIHS model was completed. Phase II was the resuscitation and rewarming period. During phase II, the rats were taken out of the seawater, and then the rats were placed in the temperature-control box (34–37°C) for resuscitation. The different osmotic pressures of resuscitation fluid including 0.9, 0.6, 0.3% saline water, and LR with 37°C were infused two times with blood loss (25 ml/h). Rats in the SIHS group were subjected to SIHS without fluid resuscitation. Rats in the sham-operated group were subjected to catheterization and seawater immersion without hemorrhage. According to the previous experimental results, the rewarming protocol was a stepped rewarming mode, that is, after the rats were weaned from seawater into the incubator, the core temperature was maintained at 34°C for 2 h and then rewarmed to 37°C. Phase III was the phase of parameter measurements. In this phase, coagulation functions, organ blood flow, organ function, mitochondrial function, the permeability of the lungs, kidneys, and intestines, the water content of the lung and brain, the MAP, and animal survival were observed (Figure 1).

### Coagulation Function Measurement

After phase II, 2 ml of blood (citric acid:blood = 1:9) was used to measure the coagulation functions using the ACLTOP700 instrument (Werfen, American), including thrombin time (TT), prothrombin time (PT), the international normalized ratio of prothrombin time (PT-INR), and activated partial prothrombin time (APTT).

### The Measurement of Blood Flow in the Vital Organs

The blood flow in the liver, kidney, and intestines was measured by a Doppler imaging instrument (Peri Cam PSI ZR, Sweden); the organ was exposed to a laser at a distance of 14 cm, the blood flow in the liver, kidney, and intestines was analyzed using the PIM software.

### The Measurement of Mitochondrial Respiratory Control Rate and ATP Content

The tissues of the heart, liver, kidneys, and intestines were used to observe the mitochondrial respiratory control rate. The method was described in our previous study (Li et al., 2012). Briefly, 5 g samples were placed in the precooled isolation buffer (sucrose 0.25 M, Na_2_EDTA 0.1 mM, Tris 0.01 M, pH 7.6) and washed until the blood was removed. The sample was fully homogenized and then centrifuged at 1,600 *g* for 12 min at 4°C. The supernatant was further centrifuged two times at 25,000 *g* for 15 min at 4°C. The precipitation was collected and resuspended with an isolation buffer. The concentration of the mitochondrial protein was measured by the Lowry method: reaction buffer (30°C, Tris 0.2 M, pH 7.6, KCl 15 mM, KH_2_PO_4_ 15 mM, Na_2_EDTA 1 mM, MgCl_2_ 5 mM, and sucrose 0.25 M) was added into the chamber until equilibrium. Then, mitochondrial mixture, sodium malate, sodium glutamate, and adenosine diphosphate were added consecutively. The rate of oxygen consumption was determined using the mitochondrial function analyzer (MT200; Strathkelvin).

The ATP content was measured according to the luciferin-luciferase method. Tissue samples were lysed using the ATP lysis buffer on ice and were assayed for ATP using a chemical luciferase ATP assay kit (Beyotime Institute of Biotechnology, https://www.beyotime.com/index.htm). The quantity of ATP was calculated from a standard curve and was expressed as nmol/mg protein.

### The Measurement of Vital Organ Function

#### Echocardiography

Echocardiography was performed in anesthetized rats using a Vivid 9 high-frequency color Doppler ultrasound system (GE Healthcare, Boston, MA, United States). Long-axis M-mode echocardiograms were used to measure the left ventricular (LV) and systolic functions, and fractional shortening (FS) and ejection fraction (EF) were calculated.

#### Biochemical Indicators

A total of 2 ml of blood was used to measure the liver function [i.e., alanine aminotransferase (ALT) and aspartate aminotransferase (AST)], renal function [blood urea nitrogen (BUN) and serum creatinine (Crea)], and cardiac function (troponin T, TnT) with the biochemical analyzer (DX800, Biochemical Analyzer; Beckman, Fullerton, CA, United States).

#### The Measurement of Vascular Permeability and Tissue Edema in Different Organs

According to the previous report, fluorescein isothiocyanate-labeled bovine serum albumin (FITC-BSA, 9 mg/kg) was injected into rats from the jugular vein. Following the circulation for 2 h, the rats were injected with 20 ml heparin sodium (5 U/kg) to flush the vessels of the lungs, kidneys, or intestines (Zheng et al., 2020). Then, the rats were sacrificed by euthanasia; the left upper lobe of the lung, left kidney, and ∼6 cm of the jejunum were separated, weighed, and cut into pieces. The tissue was homogenized and centrifuged at 8,000 *g* × 4°C for 10 min, and the supernatant was determined using the fluorescence spectrophotometer absorbance (at wavelengths of 500 and 530 nm). The fluorescence intensity of tissue supernatant (OD)/tissue weight (g) was used to represent the permeability of the lung, kidney, and intestine.

The rat was sacrificed by injection overdose of sodium pentobarbital. The brain and lung were quickly removed to measure the water content. After the tissue was separated and weighed, the tissue was dried at 105°C for 72 h, and then the dry weight was calculated. The percentage of the water content was calculated as [(wet weight–dry weight)/wet weight] × 100% (Hu et al., 2015).

#### Statistical Analyses

All data were expressed as mean ± standard deviation. The body temperature, coagulation function, liver and kidney function, and pH were measured by repeated measures one-way or two-way analysis of variance followed by the *post hoc* Tukey test (SPSS17.0; SPSS Incorporated, Chicago, IL, United States). The survival time of animals was compared between groups using the Kaplan-Meier method and the log-rank test. A *p*-value < 0.05 was considered statistically significant (two-tailed).

## Results

### Effects of Different Osmotic Fluids on Lethal Triad Following Seawater Immersion Combined With Hemorrhagic Shock in Rats

The parameters of the lethal triad included dysfunction of coagulation, acidosis, and the decrease of the body temperature; the effects of fluids with different osmotic pressures (LR, 0.3% saline, 0.6% saline, and 0.9% normal saline) on the lethal triad following SIHS in rats were observed.

#### Effects on Acidosis

**FIGURE 2:**
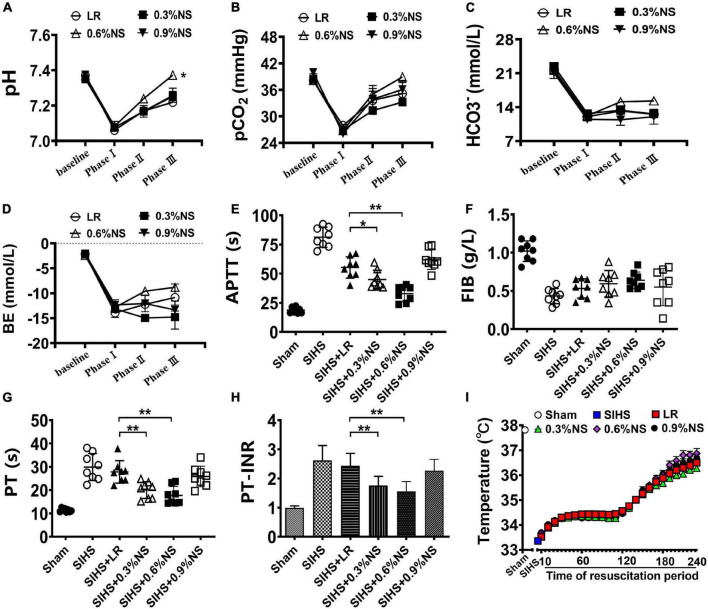
Effects of different osmotic fluids on lethal triad with seawater immersion in hemorrhagic shock rats. **(A)**: pH value of blood. **(B)** Partial pressure of carbon dioxide (PCO_2_). **(C)** Bicarbonate ion (HCO_3_^–^). **(D)** Residual base (BE). **(E)** Activated partial thromboplastin time (APTT). **(F)** Fibrinogen (FIB). **(G)** Prothrombin time (PT). **(H)** Prothrombin time-international normalized ratio (PT-INR). **(I)** Core temperature. LR, lactated Ringer’s solution; NS, normal saline; Sham, Sham-operated group; SIHS, seawater immersion combined with hemorrhagic shock; baseline, before model. Phase I, SIHS for 2 h; Phase II, at the end of resuscitation; Phase III, at the end of rewarming and recovery treatment. Temperature, the core temperature and was recorded every 10 min from the out of seawater immersion until the end of the experiment, for a total of 4 h. *p < 0.05, compared with LR control group; **p < 0.01, compared with LR control group.

Acidosis is the accumulation of acid in blood or tissues, which is considered a vital factor resulting in organ function disorder (Lim, 2007; Ahmed et al., 2019). The results showed that the pH value of blood in rats was significantly decreased to 7.06 after SIHS. Fluid resuscitation alleviated the acidosis status of the rats with different degrees. Following LR resuscitation, the pH value of rats was increased to 7.27, and the pH value after the infusion with 0.9% normal saline showed a similar trend with the LR group. The infusion of 0.6% saline significantly corrected acidosis, and the value of pH was recovered to 7.37, while 0.3% of the normal saline could not correct acidosis (Figure 2A).

Besides the pH value, the changes of PCO_2_, HCO_3_^–^, and BE were consistent with the change of the pH value, and they were decreased to 27.91 mmHg, 12.05 mmol/L, and −12.85 mmol/L, respectively, following SIHS. LR resuscitation increased the values of the PCO_2_, HCO_3_^–^, and BE to 35.18 mmHg, 12.59 mmol/L, and −5.94 mmol/L, respectively. The values of PCO_2_, HCO_3_^–^, and BE in the 0.6% saline group were greater than those in the LR group (Figures 2B–D).

#### Effects on Coagulation Function

Hemorrhagic shock combined with seawater immersion led to the disturbances in the coagulation function, including the increases of APTT, PT, PT-INR, and the decrease of FIB. Fluid resuscitation improved the coagulation disorder in rats with different degrees. The values of APTT, PT, PT-INR, and FIB in the LR group were 55.33 s, 27.81 s, 2.44, and 0.43 mg/ml, respectively, at the end of resuscitation. The values of coagulation parameters in the 0.9% normal saline group were similar to those of LR; there was no significant difference. Infusion of 0.6% normal saline significantly alleviated hemorrhagic shock combined with seawater immersion induced the increase of values of APTT, PT, and PT-INR as compared with the LR group and 0.9% normal saline group, and they almost returned to the normal level. The 0.3% normal saline could not improve coagulation function disorder as compared with the LR group (Figures 2E–H).

#### Effects on Body Temperature

Seawater immersion induced the decrease of the core temperature significantly to 33°C in rats. According to the treatment guideline of seawater immersion, the rewarming protocol was carried out. The rats were placed in a temperature-controlled chamber for rewarming and resuscitation treatment after taken out of seawater immersion. The body temperature rose to 34°C after treatment for 30 min, and then the body temperature was rewarmed to 37°C. The core temperatures of rats in each group were maintained as the designed level (Figure 2I).

### Effects of Different Osmotic Fluids on Blood Flow and Mitochondrial Function of the Vital Organs Following Seawater Immersion Combined With Hemorrhagic Shock in Rats

#### Blood Flow in Vital Organs

**FIGURE 3:**
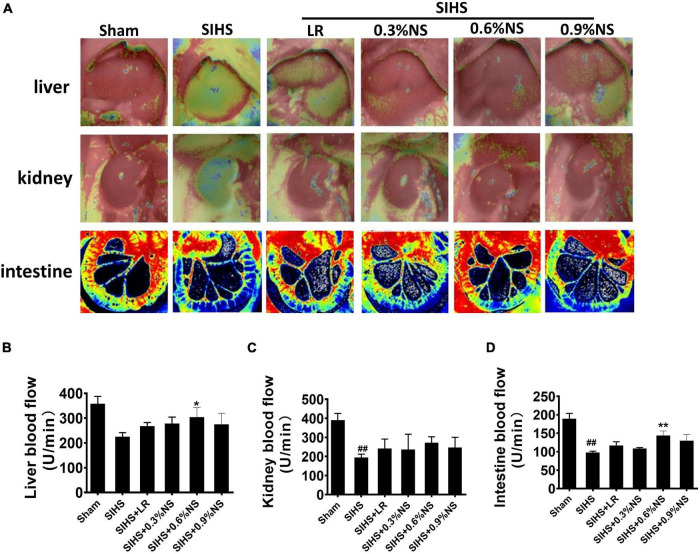
Effects of different osmotic fluids on blood flow with seawater immersion in hemorrhagic shock rats (*n* = 8/group). **(A)** Blood flow in the liver, kidney, and intestine with the Peri Cam PSI System. Blood flow in the liver, kidney, and intestine with laser Doppler imaging. **(B)** Statistical analysis of liver blood flow. **(C)** Statistical analysis of kidney blood flow. **(D)** Statistical analysis of intestinal blood flow. Sham, Sham-operated group; SIHS, seawater immersion combined with hemorrhagic shock; LR, lactated Ringer’s solution; NS, normal saline. *p < 0.05 and **p < 0.01, compared with LR group. ##*p* < 0.01, compared with the Sham group.

Previous studies showed that the perfusions of the vital organs were significantly decreased after hemorrhagic shock (Li et al., 2013). This study showed that the blood flow in the liver, kidney, and intestines was significantly decreased after SIHS. Fluid resuscitation could improve the blood flow in these organs. The effects of 0.6% normal saline group were better than other groups. The blood flow in the liver, kidney, and intestine in the 0.6% normal saline group was 303.81, 272.87, and 143.82 U/min, respectively. As compared with the LR group, the blood flow in the liver, kidney, and intestine was increased by 13.50, 45.70, and 30.33%, respectively (Figures 3A–D).

#### Mitochondrial Function of Vital Organs

**FIGURE 4:**
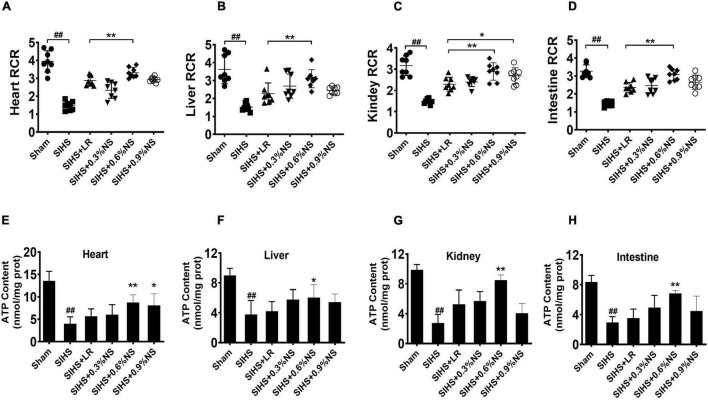
Effects of different osmotic fluids on mitochondrial function with seawater immersion in hemorrhagic shock rats (*n* = 8/group). **(A)** Mitochondrial respiratory control rate of the heart. **(B)** Mitochondrial respiratory control rate of the liver. **(C)** Mitochondrial respiratory control rate of the kidney. **(D)** Mitochondrial respiratory control rate of the intestines. **(E)** ATP contents of the heart tissue. **(F)** ATP contents of the liver. **(G)** ATP contents of the kidney. **(H)** ATP contents of the intestine. Sham, Sham-operated group; SIHS, seawater immersion combined with hemorrhagic shock; LR, lactated Ringer’s solution; NS, normal saline; RCR, respiratory control rate. * *p* < 0.05 and **p < 0.01, compared with the LR group. ##*p* < 0.01, compared with the Sham group.

Mitochondria are the major organelle of cellular aerobic respiration, providing energy for cellular metabolism (Annesley and Fisher, 2019). Mitochondrial function determines the organ function. Previous studies found that hypothermia or acidosis induced disorder of the mitochondrial function, which in turn aggravated acidosis and hypothermia. The mitochondrial function after infusion with different osmotic fluids was investigated. The results showed that mitochondrial respiration control rates in the heart, liver, kidney, and intestines significantly decreased after SIHS. Both LR and saline resuscitation of different osmotic pressures could partially restore mitochondrial respiratory control rates. The effects of 0.6% saline were better than other fluid resuscitation. Compared with LR, the mitochondrial respiratory control rates of the heart, liver, kidney, and intestine in the 0.6% normal saline group increased by 15.03, 37.20, 25.98, and 30.57%, respectively (Figures 4A–D). ATP contents in the cardiac muscle were decreased significantly after SIHS. LR could slightly improve the ATP contents; the effects of infusion with 0.6% normal saline were more obvious (Figure 4E). The trends of ATP contents in the liver, kidney, and intestine were similar to that of the heart (Figures 4F–H).

### Effects of Different Osmotic Fluids on Blood Osmotic Pressure, Vascular Permeability, and Tissue Edema of Vital Organs in Seawater Immersion Combined With Hemorrhagic Shock Rats

**FIGURE 5:**
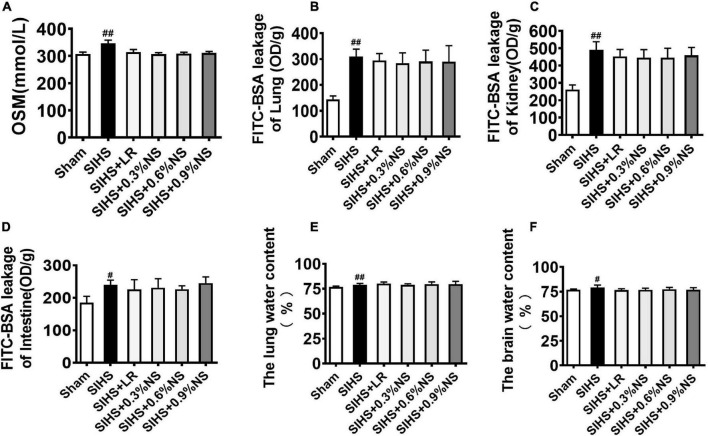
Effects of different osmotic fluids on blood osmotic pressure, vascular permeability, and tissue edema of vital organs in SIHS rats. **(A)** Plasma osmotic pressure. **(B)** Fluorescein isothiocyanate-labeled bovine serum albumin (FITC-BSA) leakage of the lung. **(C)** FITC-BSA leakage of the kidney. **(D)** FITC-BSA leakage of the intestine. **(E)** Lung water content. **(F)** Brain water content. FITC-BSA enters the blood circulation from the jugular vein and then leaks into the lungs, kidneys, and intestines. FITC-BSA leakage of lung/kidney/intestine was used to represent the permeability of lung/kidney/intestine. Sham, Sham-operated group; SIHS, seawater immersion combined with hemorrhagic shock; LR, lactated Ringer’s solution; NS, normal saline. #*p* < 0.05 and ##*p* < 0.01, compared with the Sham group.

Vascular leakage played an important role in the occurrence of organ function damage (Zhang et al., 2018). It was not clear whether different osmotic fluid could induce the change of plasma osmotic pressure, which results in the increase of the water content of tissue and organ function disorder. In this study, the effects of different osmotic pressures on the water content and vascular permeability with SIHS rats were observed. The results showed that the plasma osmotic pressure significantly increased from 307.5 to 346.7 mmol/L following SIHS. Fluid resuscitation could decrease the osmotic pressure, and there was no significant difference among different osmotic fluids (Figure 5A). The permeabilities of the lung, kidney, and intestine increased after SIHS, while no significant increase was found after fluid resuscitation with different osmotic pressures (Figures 5B–D). The water contents of the lung and brain were increased at the end of the shock, and fluid resuscitation with different osmotic pressures could decrease the water contents of the lung and brain (Figures 5E,F). The results indicate that fluids with different osmotic pressures could not induce tissue edema.

### Effects of Different Osmotic Fluids on Vital Organ Function After Seawater Immersion Combined With Hemorrhagic Shock in Rats

**FIGURE 6:**
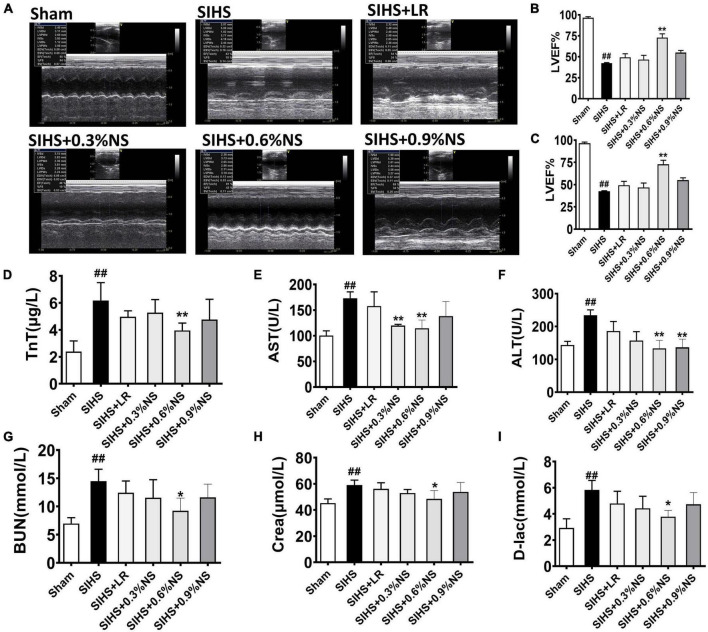
Effects of different osmotic fluids on vital organ function with seawater immersion in hemorrhagic shock rats (*n* = 8/group). **(A)** Echocardiograms. **(B–C)** Summary of cardiac ejection fraction (EF) **(B)** and fractional shortening (FS) **(C)** measured using echocardiography. **(D)** Troponin T (TnT). **(E)** Aspartate transaminase (AST). **(F)** Alanine aminotransferase (ALT). **(G)** Urea nitrogen (BUN). **(H)** Creatinine (Crea). **(I)** D-Lactate. Sham, Sham-operated group; SIHS, seawater immersion combined with hemorrhagic shock; LR, lactated Ringer’s solution; NS, normal saline. *p < 0.05 and **p < 0.01, compared with the control group. ##*p* < 0.01, compared with the Sham group.

Organ dysfunction played an important role in the occurrence and development of hemorrhagic shock and was the important inducer of death in shock patients (Torres Filho, 2017). Echocardiography was used to evaluate the cardiac function. The results showed that the cardiac function was significantly impaired after SIHS, which was presented with a decrease of LVEF and LVFS. 0.6% normal saline infusion significantly improved the systolic and diastolic functions of rats, and significantly increased LVEF and LVFS (Figures 6A–C). Damage indicators of the heart, liver, kidney, and intestines were observed in SIHS rats. The results showed that the functions of the heart, liver, kidney, and intestines were significantly impaired after SIHS, and the indexes of heart damage (i.e., TnT), liver indexes (i.e., AST and ALT), kidney damage indexes (i.e., BUN and Crea), and intestines damage indexes (i.e., D-Lac) were significantly increased. Different resuscitation fluids could partially alleviate the vital organ dysfunction in rats. The levels of TnT, AST, ALT, BUN, Crea, and D-Lac in the LR group were 4.97 μg/L, 157.70 U/L, 185.96 U/L, 12.44 mmol/L, 50.00 μmol/L, and 4.80 mmol/L, respectively. As compared with the LR group, the levels of TnT, AST, ALT, BUN, and D-Lac in the 0.6% normal saline group were decreased by 21.36, 27.37, 28.36, 25.82, and 21.07%, respectively (Figures 6D–I).

### Effects of Different Osmotic Fluids on Survival Following Seawater Immersion Combined With Hemorrhagic Shock in Rats

**FIGURE 7:**
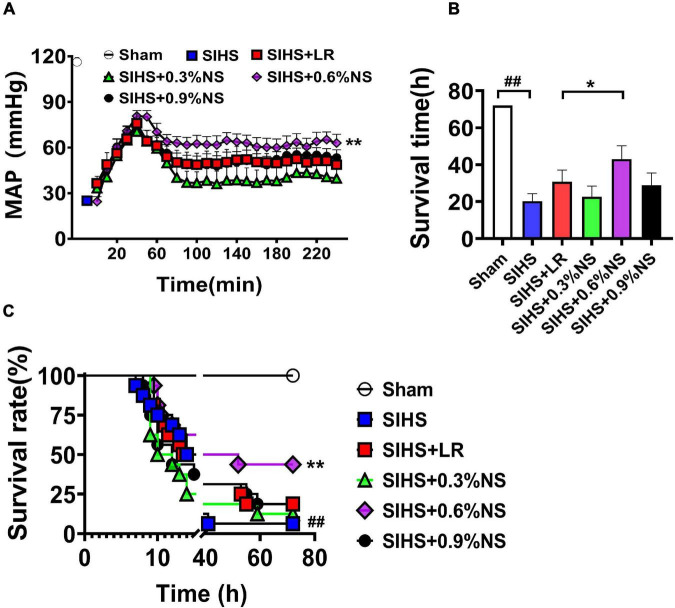
Effects of different osmotic fluids on mean arterial pressure and survival with seawater immersion in hemorrhagic shock rats. **(A)** Mean arterial pressure (MAP) (*n* = 8/group). **(B)** Survival time (*n* = 16/group). **(C)** Survival rate (*n* = 16/group). MAP, mean arterial pressure; Sham, Sham-operated group; SIHS, seawater immersion combined with hemorrhagic shock; LR, lactate Ringer’s solution; NS, normal saline. *p < 0.05 and **p < 0.01, compared with the LR control group. ##*p* < 0.01, compared with the Sham group.

The above results indicated that fluid resuscitation with different osmotic pressures could improve the vital organ function at SIHS rats with different degrees. Animal survival and mean arterial pressure (MAP) were further observed. The results showed that MAP was significantly decreased to 30 mmHg after SIHS. Fluid resuscitation with different osmotic pressures could increase MAP to 80 mmHg at the beginning of the resuscitation. While MAP could not be maintained at this level with different osmotic pressures, MAP fell to the shock level in the 0.3% normal saline group. The MAP was decreased to 60 mmHg in the 0.9% normal saline group and LR group. The MAP in the 0.6% normal saline group was maintained at 60–70 mmHg (Figure 7A).

The results of the survival study showed that only one rat survived to 72 h after SIHS without resuscitation; the average survival time was 20.31 ± 15.64 h. Following LR resuscitation, the survival times and 72 h survival rate were 30.78 ± 24.35 h and 3/16, respectively. There were 2, 7, and 3 rats that survived till 72 h in 0.3, 0.6, and 0.9% normal saline groups; the survival times were 22.69 ± 22.37 h, 43.03 ± 27.81 h, and 28.91 ± 25.62 h, respectively (Figures 7B,C).

## Discussion

Our previous studies demonstrated that the pathophysiological parameters after seawater immersion were mainly characterized by a high incidence of the lethal triad, electrolyte disturbance, severe organ dysfunction, and a high mortality rate. There are no effective measures, and which fluid was suitable for the treatment at the condition of SIHS is not known. In this study, we found that mild hypotonic fluid resuscitation was suitable for the early treatment of SIHS; as compared with LR or normal osmotic fluid, it alleviated the acidosis and coagulation dysfunction, protected the organ function, prolonged the survival time of animals, and improved the survival rate. This study provided optional fluid for hemorrhagic shock in combination with seawater immersion. Osmotic pressure modulated the cell functions, including the cell growth. Heimer showed that hyperosmotic pressure inhibited the cell proliferation and promoted cell death. Besides cell proliferation, previous studies found that a low osmotic pressure led to mitochondrial damage (Heimer et al., 2018). And Shabalina demonstrated that ATP contents of mitochondria were significantly increased following hypotonic medium incubation (Shabalina and Panov, 1991). In this study, we found that mild hypotonic fluid improved mitochondrial ATP contents and OCR and then improved organ functions. About the mechanisms of osmotic pressure regulating mitochondria, there were few reports. Chen found that appropriate hypotonic stress (225 mOsm.kg/H_2_O) could stabilize mitochondrial membrane potential and reduce the release of cytochrome C from mitochondria to the cytoplasm by increasing the Bcl-2/Bax ratio, thus inhibiting the mitochondria-dependent apoptotic pathway (Chen et al., 2020). Qian et al. (2011) found that hypotonic-induced volume-regulating Cl^–^ channels (VRCCs) had a protective effect on H_2_O_2_^–^-induced apoptosis in smooth muscle cells. Following seawater immersion and hemorrhagic shock, the rats were in a hypertonic environment, and the ion concentration in plasma was increased. Appropriate hypotonic might reopen VRCCs and then lead to Cl^–^ effusion, reduced intracellular Cl^–^ concentration, stabilized mitochondrial membrane potential, and mitochondrial function. But the specific mechanism needs to be further studied.

In this study, the damage indexes of the liver and kidney functions were significantly increased, and organ functions were seriously impaired after SIHS. After treatment with fluids with different osmotic pressures, the damage of the liver and kidney function was lessened, among which 0.6% saline group had the best effect. These results indicated that mild hypotonic effects were beneficial on the liver and kidney function of rats with SIHS. The mechanisms may be related with that during the plasma osmotic pressure of the rats increasing, resuscitation with isotonic fluid would result in hemolysis and aggravate organ function damage (Van Regenmortel et al., 2017). The other reason may be related with that the isosmotic fluid had a longer metabolic time than the hypotonic solution, which resulted in the increase of chloride ions in blood circulation and then led to hyperchloremia and aggravated the burden of the kidney (Blumberg et al., 2018). Moreover, our study also showed that too low osmotic pressure fluid resuscitation led to further organ function disorder. The detail mechanisms are not clear. Previous studies showed that fluid resuscitation with too low osmotic pressure could induce various degrees of damage to glial cells and lead to changes in cell function and metabolism (Shiozaki et al., 2014). Wang et al. (2013) also found that mild hypotonic solution induced adaptive changes in the osmotic pressure of neurons and prevented the occurrence of disseminated nodules in gastric cancer cells (Shiozaki et al., 2014). The detail mechanisms of fluid resuscitation with different osmotic pressures on organ function need further study.

This study showed that the rats were suffered from severe acidosis after being immersed in seawater and hemorrhagic shock. Previous studies found that the microcirculation was inadequately perfused during SIHS; the intestinal barrier function was weakened and then resulted in acidosis (Rae et al., 2016). This may be a major reason that was related with SIHS. This study found that fluid resuscitation with different osmotic pressures improved the pH value with SIHS rats. It had been reported that isotonic solution of 0.9% normal saline led to metabolic acidosis (Blumberg et al., 2018). Our study also found that compared with the group of 0.6% saline, the pH value was decreased even more dramatically, and the recovery was slower after the treatment, and organ function damage was more serious in the group of 0.9% normal saline. The hyperventilation led to the decrease of PCO_2_ after hemorrhagic shock; meanwhile, the hypothermia led to the retention of CO_2_ and the decrease of HCO_3_^–^ during seawater immersion (Kerger et al., 1999). The double blow made the acidosis worse. After treatment with fluids with different osmotic pressures, PCO_2_ and HCO_3_^–^ were increased to some extent. It was indicated that the different osmotic pressures of fluids were beneficial to correct the acidosis caused by hemorrhagic shock combination with seawater immersion.

The electrolyte and plasma osmotic pressure of the body were significantly disordered after seawater immersion, which was a vital factor inducing a high mortality rate (Huang et al., 2010). The osmotic pressure of seawater was (1,250 ± 1.52) mmol/L, the concentration of sodium ion was 0.625–0.635 mol/L, and the pH was 8.2 (Shen et al., 2017). Following SIHS, severe pathophysiological parameters such as hypovolemia and hyperammonemia occurred in the body. Therefore, replenishing blood volume and effectively correcting the hyperosmolarity state of the body were key to early resuscitation. In this study, appropriate hypotonic fluid was used to replenish the blood volume. Previous study also found that appropriate hypotonic sodium chloride could relieve lung and brain edema and had a good effect on correcting hyperosmolarity in seawater immersion injury of the thoracic cavity (Li et al., 2001). Our results further confirmed that appropriate hypotonic could improve the lethal triad and protect the organ function. The protective effect of hypotonic fluids on organ functions might be due to the fact that the concentration of sodium chloride in the hypotonic fluids could slowly reduce the osmotic pressure, thus avoiding tissue edema and cell edema caused by too low osmotic pressure drop.

Previous studies demonstrated that patients with low temperature immersed in seawater had long been considered as rapid rewarming, but rapid rewarming could lead to rewarming shock and more serious injury to patients. Research showed that the hypothermia rats were rewarmed at 37°C for 1 h, and the organ function of the rats was significantly improved, but the duration time of 37°C needed to be strictly controlled, and complications still occurred after the body temperature returned to normal (Huan et al., 2018). In the previous experiment, we compared the rapid rewarming and step-up rewarming and found that the effects of step-up rewarming were significantly better than that of rapid rewarming (data not listed). Therefore, in this experiment, step-up rewarming was adopted, that is, when the rats were out of the low-temperature seawater, they were immediately placed in the 37°C temperature-controlled chamber. When the core temperature reached 34°C, the heating was stopped immediately and the ambient temperature was reduced to 25°C. At this time, the core temperature of the rats could be maintained at 34°C for 2 h, and then the ambient temperature was heated to 37°C for 2 h.

Although this study showed that mild hypotonic fluid was a suitable therapeutic fluid in hemorrhagic shock rats with seawater immersion, there are still some shortcomings. First, this experiment only observed the effects of fluids with different osmotic pressures on the liver and kidney, and the changes of other organs were not observed; Second, the study only observed the therapeutic effects of different osmotic fluids in hemorrhagic shock rats with seawater immersion, but its mechanism and side effects were not clear, which will be the direction of our future work.

## Conclusion

In summary, mild hypotonic fluid is suitable for early resuscitation after SIHS by alleviation of acidosis and coagulation dysfunction, resulting in the improvement of organ functions and the survival of animals. Moderate hypotonic fluid would be beneficial to the treatment of SIHS.

## Data Availability Statement

The original contributions presented in the study are included in the article/supplementary material, further inquiries can be directed to the corresponding author/s.

## Ethics Statement

The animal study was reviewed and approved by Research Council and Animal Care and Use Committee of Research Institute of surgery, Army Medical University.

## Author Contributions

TL and LL designed the study. JZ, YW, HS, and YQZ analyzed the data. TL and HD acquired the financial support. YZ drafted the manuscript. HD revised the manuscript. All authors performed the experimental procedures.

## Conflict of Interest

The authors declare that the research was conducted in the absence of any commercial or financial relationships that could be construed as a potential conflict of interest.

## Publisher’s Note

All claims expressed in this article are solely those of the authors and do not necessarily represent those of their affiliated organizations, or those of the publisher, the editors and the reviewers. Any product that may be evaluated in this article, or claim that may be made by its manufacturer, is not guaranteed or endorsed by the publisher.
